# *In vivo* retention and bioactivity of IL-1ra microspheres in the rat intervertebral disc: a preliminary investigation

**DOI:** 10.1186/s40634-014-0015-8

**Published:** 2014-11-21

**Authors:** Deborah J Gorth, John T Martin, George R Dodge, Dawn M Elliott, Neil R Malhotra, Robert L Mauck, Lachlan J Smith

**Affiliations:** Department of Neurosurgery, Perelman School of Medicine, University of Pennsylvania, 424 Stemmler Hall, 3450 Hamilton Walk, Philadelphia, PA 19104 USA; Department of Orthopaedic Surgery, Perelman School of Medicine, University of Pennsylvania, 424 Stemmler Hall, 3450 Hamilton Walk, Philadelphia, PA 19104 USA; Translational Musculoskeletal Research Center, Veterans Affairs Medical Center, 3900 Woodland Avenue, Philadelphia, PA 19104 USA; Department of Biomedical Engineering, College of Engineering, University of Delaware, 125 E. Delaware Ave, Newark, DE 19716 USA

**Keywords:** Intervertebral disc degeneration, Inflammation, Interleukin-1 receptor antagonist, PLGA microspheres, Rat, *In vivo*

## Abstract

**Background:**

Inflammatory cytokines such as interleukin-1 beta (IL-1β) contribute to the progression of intervertebral disc degeneration. Previously we demonstrated, *in vitro*, that by delivering interleukin-1 receptor antagonist (IL-1ra) from poly(lactic co-glycolic acid) (PLGA) microspheres, we could attenuate the degradative effects of IL-1β on the nucleus pulposus (NP) for up to 20 days. The objective of this study was to undertake a preliminary investigation into whether microspheres could be successfully delivered to and retained in the disc *in vivo*, and whether IL-1ra released from those microspheres remained biologically active. For retention studies, fluorescently-labeled microspheres were delivered to the NPs of rat caudal discs. Rats were sacrificed at time points up to 56 days, and microspheres were localized using fluorescent microscopy. To investigate whether IL-1ra microspheres could effectively inhibit the effects of IL-1β *in vivo*, four disc levels were allocated to the following treatment groups: intact; saline; IL-1β; or IL-1β + IL-1ra microspheres. Rats were sacrificed after seven days and NP glycosaminoglycan content was measured.

**Findings:**

Microspheres were visible in the disc at all time points up to 28 days, and localized to the NP, the annulus fibrosus (AF), or both. Glycosaminoglycan content for discs injected with IL-1β alone was significantly lower than for intact controls. For discs injected with IL-1β along with IL-1ra microspheres, glycosaminoglycan content was not significantly different from intact controls.

**Conclusions:**

Microspheres can successfully be delivered to the disc *in vivo* and retained for a clinically relevant time frame. IL-1ra released from microspheres can effectively prevent IL-1β-induced NP glycosaminoglycan loss *in vivo*.

## Introduction

Intervertebral disc degeneration is a cascade beginning with changes to the local cellular microenvironment and progressing over years to structural breakdown and functional impairment [1,2]. Changes to the central nucleus pulposus (NP) are implicated in the early stages of disc degeneration, where decreasing proteoglycan content and an associated reduction in hydrostatic pressure impair the ability of the disc to evenly distribute and transfer compressive loads between the vertebrae [3]. Inflammation plays a key role in the progression of disc degeneration. Interleukin-1 beta (IL-1β), in particular, is more highly expressed in the degenerate NP than in the healthy NP [4]. Elevated IL-1β induces catabolic protease activity, which degrades the disc extracellular matrix and contributes to structural failure [5]. Interleukin-1 receptor antagonist (IL-1ra) is an endogenous inhibitor of IL-1β, where it acts by competitively blocking the binding of IL-1β to receptors on IL-1 responsive cells [6]. While IL-1β is upregulated with disc degeneration, there is no concomitant upregulation of IL-1ra [4], resulting in an imbalance between catabolic and anabolic signaling. In our previous *in vitro* work, we demonstrated that soluble IL-1ra effectively inhibited the degradative effects of IL-1β on NP cells [7]. Further, by delivering IL-1ra from poly(lactic-co-glycolic acid) (PLGA) microspheres, inhibition could be maintained for up to 20 days [8]. Key advantages of PLGA microspheres for drug delivery include that they are biodegradable and biocompatible, already FDA approved for other clinical indications, and most importantly, can be easily tuned to alter degradation and protein release kinetics [9,10].

The objective of this study was to advance this encouraging *in vitro* work to towards clinical translation, by undertaking a preliminary *in vivo* study to demonstrate, firstly, that PLGA microspheres could be delivered to the disc and retained for a clinically relevant time frame *in vivo*; and, secondly, that IL-1ra delivered from these microspheres maintained bioactivity in the *in vivo* space.

## Materials and methods

### Microsphere fabrication

PLGA microspheres were fabricated as described previously, using the water/oil/water double emulsion technique [8,9]. As in our previous work, we used the pharmaceutical form of IL-1ra, anakinra (Amgen, Thousand Oaks, CA). The initial water/oil emulsion was formed by combining 225μL of IL-1ra (0.15 g/ml) with 1 ml of 75:25 PLGA (0.1 g/ml) (Durect Corporation, Pelham, AL). For fluorescent microsphere studies, the PLGA was supplemented with 3 mg/ml coumarin-6 (Sigma Aldrich; λ_emission_ =505 nm; λ_absorption_ =444 nm), and saline was substituted for IL-1ra. Fabricated microspheres were lyophilized until required for surgeries, and sterilized via exposure to ultraviolet light for 10 min prior to injection. The release profile of IL-1ra from these microspheres was described previously, and is characterized by an initial burst release, decreasing to an approximately linear and sustained release over the initial 10 days. *In vitro*, bioactive IL-1ra continues to be released for up to 20 days [8].

### In vivo microsphere retention study

**Figure 1:**
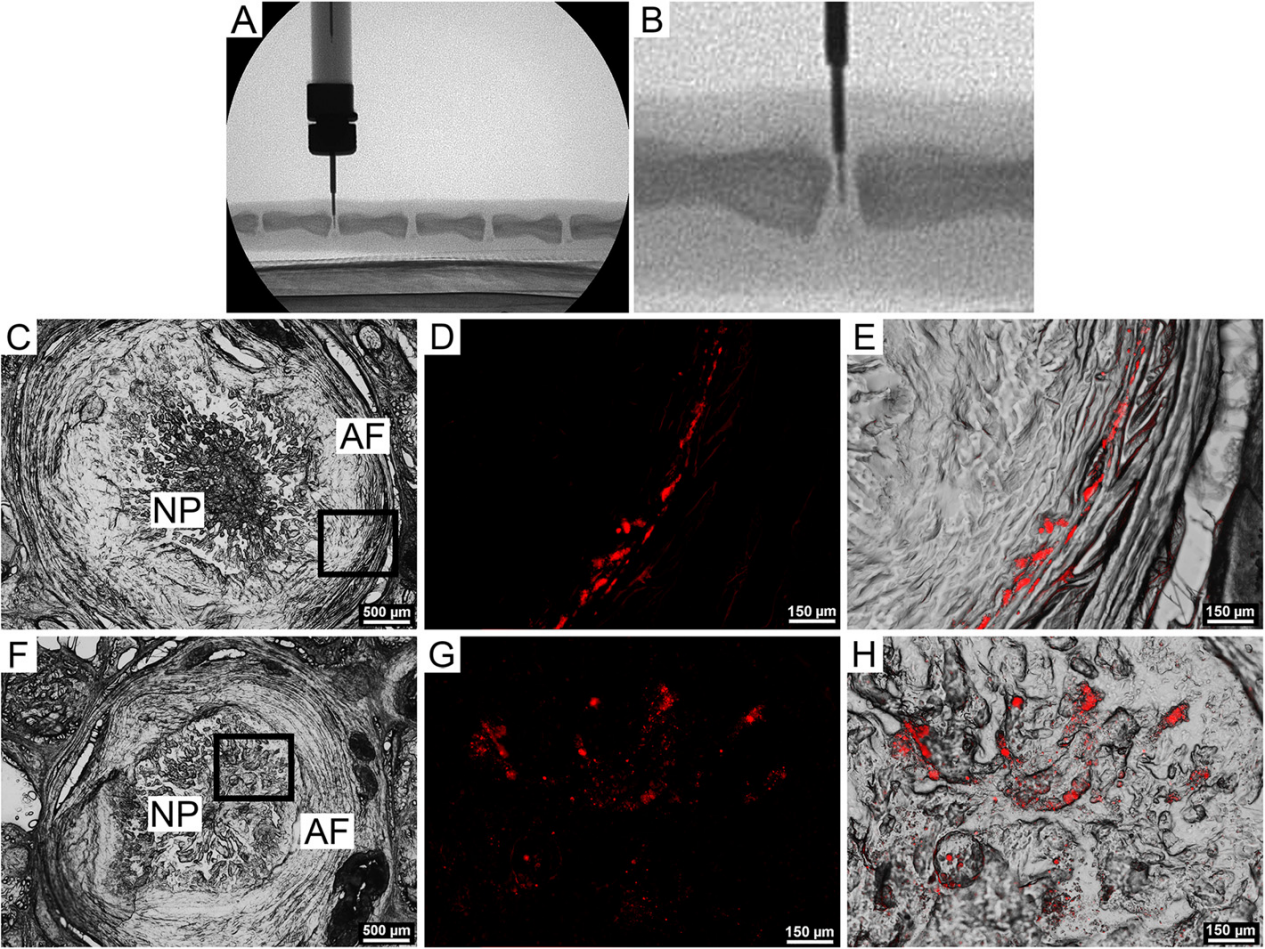
***In vivo*** **delivery and localization microspheres. A**. Delivery of microspheres to the rat NP from a custom 33-gauge needle under fluoroscopic guidance (lateral aspect). **B**. Higher magnification view of A. **C**. Differential interference contrast (DIC), axial image of entire disc 1 day post injection, showing NP and AF regions. **D**. Higher magnification view of the AF (inset from C.) showing microspheres (red). **E**. Microspheres (red, overlaying DIC image) embedded in the inner lamellae of the AF. **F**. Differential interference contrast, axial image of entire disc 14 days post injection, showing NP and AF regions. **G**. Higher magnification view (inset from F.) showing microspheres (red). **H**. Microspheres (red, overlaying DIC image), embedded in the NP.

The Animal Studies Subcommittee of the Philadelphia VA Medical Center approved all animal studies. We first performed a study to confirm that microspheres could be successfully delivered to the rat caudal intervertebral disc, and retained for a clinically relevant time period. Six, male, retired breeder, Sprague-Dawley rats aged 8 months were anesthetized via isoflurane inhalation. Caudal disc levels for injection (C7-8, C8-9 and C9-10) were identified fluoroscopically (Orthoscan HD; Orthoscan, Scottsdale, USA) by counting spine levels caudally from the sacrum. Each disc was exposed via a 5 mm dorsal incision through the skin. Under fluoroscopic guidance, fluorescent microspheres (20 mg/ml in sterile saline, total volume 1.5 μl) were injected into the NP of each of the three disc levels using custom 33-gauge needles designed to restrict the depth of needle insertion to 2.5mm (Figures 1A and B). Each injection was performed carefully over the course of 2-3 s. Animals were euthanized via carbon dioxide inhalation at each of 6 time points post-surgery: 1, 3, 7, 14, 28 and 56 days. Following euthanasia, spine segments containing injected discs were isolated from the tail by bisecting the adjacent vertebral bodies. These segments were snap frozen and sectioned (undecalcified) progressively through the vertebral bone on a cryostat microtome in the transverse plane until the disc was exposed. Sections 20 μm thick were then obtained through the entire thickness of the disc, and captured on glass microscope slides. Sections were immediately imaged on a fluorescence microscope (Eclipse 90i; Nikon, Tokyo, Japan) to determine the presence or absence of microspheres, and their location in the disc (NP or annulus fibrosus (AF)) by overlaying fluorescence images on differential interference contrast (DIC) images of the same region.

### In vivo bioactivity study

Next, we performed a study to confirm the *in vivo* bioactivity of IL-1ra microspheres delivered to the rat NP. Four caudal disc levels per animal were utilized (C7-8, C8-9, C9-10 and C10-11), and randomly allocated to one of four study groups: intact (non-injected) controls; sham (vehicle only) injection; IL-1β injection; or IL-1β + IL-1ra microspheres (20 mg/ml) injection. The study was performed for each of two IL-1β doses: 100 ng and 500 ng (each n = 4 animals). All injection volumes were 1.5 μl with sterile saline as the vehicle, delivered using modified 33-gauge needles. Surgeries were performed as described above. All animals were euthanized 7 days post-surgery. Following euthanasia, spine segments containing injected or control discs were isolated, and NP regions analyzed for GAG content. To overcome the challenges associated with accurate determination of GAG content in very small tissue specimens, we developed a dissection technique to accurately isolate NP samples of known volume. Spine segments were snap frozen and mounted in a cryostat microtome. Sections of vertebral bone were progressively removed until the transverse face of the disc was exposed. Four 100 μm-thick sections of disc were then cut and transferred, frozen, to a glass microscope slide. A 1 mm biopsy punch was then used to isolate the NP from the AF. Isolated NP samples were then digested in papain and assayed to determine total sulfated GAG content (expressed as absolute GAG per sample) using the dimethylmethylene blue technique [11]. Differences in GAG content between groups were established using ANOVAs followed by post-hoc, pairwise Tukey’s tests. Results were expressed as mean ± standard deviation, with significance defined as p < 0.05.

## Findings

### In vivo retention of microspheres

To determine whether microspheres could be successfully delivered to the rat disc and retained for a clinically relevant time frame, fluorescent microspheres were injected into rat caudal discs, and their presence or absence and localization (AF or NP) investigated at six post-surgical timepoints up to 8 weeks. Fluorescence microscopy confirmed the presence of microspheres in all discs and at all time points up to 28 days post-surgery. In discs where microspheres were present, their localization varied between the AF only (63% of discs, Figures 1C-E), the NP only (13% of discs, Figures 1F-H), or both the NP and the AF (25% of discs). There was no apparent association between time point and location of microspheres in the disc. Cryostat sections were also examined for evidence of microsphere leakage along the injection track, but no such leakage was observed.

### In vivo bioactivity of IL-1ra microspheres

**Figure 2:**
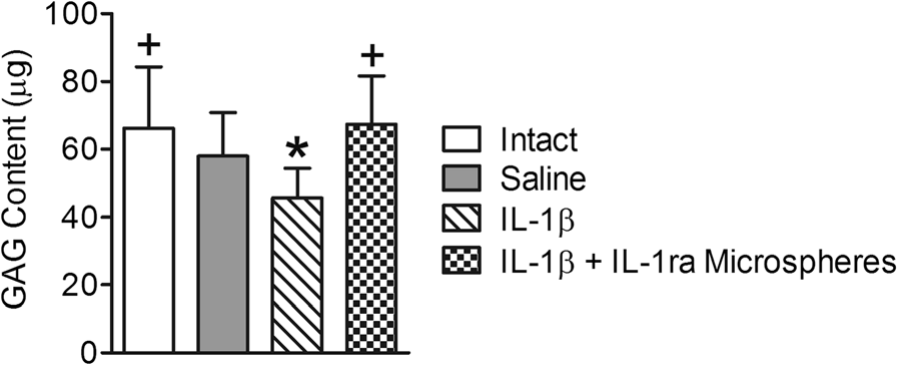
***In vivo*** **bioactivity of microspheres.** IL-1β injection alone resulted in a significantly lower NP GAG content compared to intact controls. For the combined IL-1β and IL-1ra microspheres injection, NP GAG content was not significantly different from intact controls. For the saline sham injection, NP GAG content was lower than intact controls, but not significantly, and not significantly different from IL-1β alone or combined IL-1β and IL-1ra microspheres. *p < 0.05 vs. intact controls; +p < 0.05 vs. IL-1β alone.

To confirm that the bioactivity of IL-1ra released from microspheres was maintained following *in vivo* delivery, we investigated the efficacy of IL-1ra microspheres in mitigating GAG loss induced by injection of exogenous IL-1β into rat caudal discs. No statistically significant effect of IL-1β dosage (100 ng or 500 ng) on GAG content was found, so results for both dosages were pooled for subsequent analyses. IL-1β injection alone (Figure 2), resulted in a significantly lower NP GAG content compared to intact controls (65% of intact, p < 0.05). For the combined IL-1β and IL-1ra microspheres injection, NP GAG content was maintained, and not significantly different from intact controls (102% of intact, p > 0.05). For the saline sham injection, NP GAG content was lower (87%) than intact, but not significantly, and not significantly different from IL-1β alone or combined IL-1β and IL-1ra microspheres.

## Discussion and conclusions

IL-1ra has successfully been used clinically for the treatment of rheumatoid arthritis for a number of years [12], and has recently been investigated as a potential treatment for osteoarthritis [13]. In previous work we demonstrated that soluble IL-1ra can effectively inhibit the degradative effects of IL-1β on the NP at the mechanical, compositional and molecular levels [7]. Another study showed that IL-1ra delivered both directly in solution and by gene therapy inhibited mRNA expression and activity of catabolic enzymes upregulated by IL-1β in disc tissue explants [14]. Further, IL-1ra knockout mice exhibit degenerative changes consistent with human disc degeneration [15].

The avascular nature of the disc, along with the short half-life and required high therapeutic concentrations of IL-1ra [16], suggest that *in vivo*, systemic delivery for treatment of disc degeneration is unlikely to be successful. Repeated intradiscal injections of IL-1ra that would be necessary to sustain high local concentrations, are impractical and may induce further degenerative changes via structural damage to the AF resulting from repetitive delivery. Therefore, to enhance the therapeutic efficacy of IL-1ra for treating disc degeneration, we developed a sustained release delivery vehicle for IL-1ra. Using biodegradable PLGA microspheres, we demonstrated previously that, *in vitro*, we could inhibit the degradative effects of IL-1β on the NP for up to 20 days. Building on these results, in this study we demonstrated that PLGA microspheres can be delivered to the disc *in vivo* and be retained for up to 28 days. Importantly, retention time in the disc (analogous to the time it takes for microspheres to fully degrade), is longer than the time period over which microspheres continue to release active IL-1ra at therapeutic concentrations [8]. In our microsphere retention study, variability was apparent with respect to the location of microspheres in the disc (NP, AF or both). One possible explanation for this is that, with time, microspheres diffuse away from the NP towards the AF where they are caught in the dense, fibrocartilaginous lamellae. However, as there was no apparent association between time point and location of microspheres in the disc, a more likely explanation may have been minor inconsistencies in injection location. We hypothesize that active IL-1ra released from microspheres is likely to diffuse throughout the entire disc structure, irrespective of the location of microspheres. The results of the *in vivo* bioactivity study reported here support this contention: IL-1ra microspheres successfully inhibited NP GAG loss induced by IL-1β.

This study represents a preliminary investigation into the *in vivo* efficacy of using anti-inflammatory microspheres in the intervertebral disc, and is not without limitations. Additional study groups should be investigated as part of future work, such as injection of blank (non-IL-1ra loaded) microspheres, specifically to determine if acidic microsphere degradation products exert any inflammatory effect. Clearance of these degradation products must occur via passive diffusion to vasculature outside the NP. Our previous *in vitro* work suggests that any such effects are likely quite mild [8]. Future studies should also confirm that these microspheres are active for long periods *in vivo*, in the presence of chronic inflammation, and are more effective under such circumstances than a single, bolus injection of IL-1ra. The small sample size was an addition limitation of this study, although it was sufficient to detect significant changes in GAG content.

Attenuation of inflammation in the degenerate disc is only one component of a potential biological therapy for disc degeneration. A stem cell-based component may be required to potentiate tissue regeneration. In addition to preventing further degenerative changes, IL-1ra microspheres may foster a microenvironment that is anti-inflammatory and more conducive to stem cell survival, differentiation and biosynthesis [17]. Pursuant to the encouraging findings reported here, we are now designing combined anti-inflammatory and stem cell-based therapeutics for disc regeneration and evaluating their efficacy in large animal models.

### Ethics board approval

This work was conducted with the approval of the Animal Ethics Subcommittee of the Philadelphia VA Medical Center, project number 01251.
